# Definitions and methods of measuring and reporting on injurious falls in randomised controlled fall prevention trials: a systematic review

**DOI:** 10.1186/1471-2288-12-50

**Published:** 2012-04-17

**Authors:** Michael Schwenk, Andreas Lauenroth, Christian Stock, Raquel Rodriguez Moreno, Peter Oster, Gretl McHugh, Chris Todd, Klaus Hauer

**Affiliations:** 1 Department of Geriatric Research, AGAPLESION Bethanien-Hospital/Geriatric Center at the University of Heidelberg, Rohrbacherstr.149, Heidelberg 69126, Germany; 2 Network Aging Research, University of Heidelberg, Heidelberg, Germany; 3 Division of Clinical Epidemiology and Aging Research, German Cancer Research Center Heidelberg, Heidelberg, Germany; 4 School of Nursing, Midwifery and Social Work and Manchester Academic Health Sciences Centre, University of Manchester, Manchester, UK

**Keywords:** Systematic review, Injurious falls, Elderly, Fall-related outcomes, Fall prevention trials

## Abstract

**Background:**

The standardisation of the assessment methodology and case definition represents a major precondition for the comparison of study results and the conduction of meta-analyses. International guidelines provide recommendations for the standardisation of falls methodology; however, injurious falls have not been targeted. The aim of the present article was to review systematically the range of case definitions and methods used to measure and report on injurious falls in randomised controlled trials (RCTs) on fall prevention.

**Methods:**

An electronic literature search of selected comprehensive databases was performed to identify injurious falls definitions in published trials. Inclusion criteria were: RCTs on falls prevention published in English, study population ≥ 65 years, definition of injurious falls as a study endpoint by using the terms "injuries" and "falls".

**Results:**

The search yielded 2089 articles, 2048 were excluded according to defined inclusion criteria. Forty-one articles were included. The systematic analysis of the methodology applied in RCTs disclosed substantial variations in the definition and methods used to measure and document injurious falls. The limited standardisation hampered comparability of study results. Our results also highlight that studies which used a similar, standardised definition of injurious falls showed comparable outcomes.

**Conclusions:**

No standard for defining, measuring, and documenting injurious falls could be identified among published RCTs. A standardised injurious falls definition enhances the comparability of study results as demonstrated by a subgroup of RCTs used a similar definition. Recommendations for standardising the methodology are given in the present review.

## Background

At least 30% of persons aged over 65 years experience one or more falls each year [1,2] and this proportion increases to 40% after age of 75 [3]. Both the incidence of falls and the severity of complications stemming from a fall increase with age, level of disability, and extent of functional impairment [4,5]. Falls are a major health problem in older adults, causing fall-related sequelae such as fractures, head injuries, and post-fall anxiety [6-11]. Older adults are hospitalised for fall-related injuries five times more often than they are for injuries from other causes [6]. The costs of injurious falls substantially burden health care systems [12].

The prevention of falls in older people is an important health target in many countries [13,14] and numerous studies have been published to identify appropriate intervention strategies [7,15-18]. Systematic reviews of RCTs of fall prevention interventions demonstrate that the risk of falling can effectively be reduced [7,15-18]. However, sound evidence for the reduction of injurious falls remains limited [13,16,19-23]. Evidence for the prevention of fall-related injuries has been shown in controlled trials [21,23,24] but so far no randomised controlled trial (RCT) evidence of effectiveness is available [14]. Most RCTs are underpowered to detect a significant reduction in injurious fall rates owing to the relative infrequency of injurious fall events [19,22,25] and the extremely large sample sizes required to achieve adequate statistical power.

However, severe falls and their resultant injuries have a high impact on medical sequelae [26], quality of life [27] and cost to health services [12,28]. Reducing injurious falls should therefore represent a major goal of fall prevention policy [13] and necessarily be benchmarked by a high level of evidence [29].

Because of the high cost of performing RCTs to evaluate the effects of specific fall prevention strategies on injurious falls, the combination of trial data in a meta-analysis is an attractive option but is dependent on the comparability of interventions and outcome measures. A substantial precondition for high-quality meta-analysis is a standardised methodology [30] characterised by consistency in defining injurious falls and methods of collecting and documenting falls data. However, existing international guidelines for the conduct of fall prevention trials [13] do not provide sufficient recommendations for the standardisation of injurious falls methodology. A systematic literature review on definitions and methods of measuring falls in randomised controlled fall prevention trials [31] provided a methodological consensus for defining and collecting falls, but not injurious falls, data.

Limited standardisation in defining injuries has repeatedly been reported as a serious methodological pitfall when comparing study outcomes [13,31] and has been identified as a substantial methodological challenge for future studies [32]. Standardised methodology has therefore been repeatedly requested [13,33]. However, to our knowledge, there is no systematic review focusing on injurious falls methodology in fall prevention trials aiming to develop a methodological consensus for future studies.

We therefore conducted a systematic review of definitions and methods of measuring and reporting on injurious falls in randomised controlled fall prevention trials. The aim of this study was to collect and compare definitions of injurious falls and associated assessment methods and develop an outcome data set for use in future fall prevention trials including injurious falls as a study endpoint.

## Methods

This review is part of a series of reviews on behalf of the Prevention of Falls Network Europe (ProFaNE) which evaluate methodology in RCTs of fall prevention interventions in older adults. Inclusion criteria were (1) RCTs of fall prevention interventions published in English, (2) target population ≥ 65 years, (3) definition of injurious falls as a study endpoint by using the terms "injury" and "falls".

### Search strategy

An electronic literature search was performed through Ovid MEDLINE, Ovid EMBASE, CINAHL, PsycINFO, the Cochrane library and GeroLit (all from inception until 16^th ^of July 2011). The following search strategy was applied: ("Accidental Falls/" OR "fall.mp." (mp)) AND ("Accident Prevention/" OR "prevent$.mp." OR "prophyl$.mp.") AND ("clinical trials/" OR "clinical trial.mp" OR "randomised.mp" OR "randomized.mp" OR "randomly.mp"). Reference lists of included studies and of related reviews were searched for potentially eligible studies. Moreover researchers in the field were contacted and asked for additional trial reports.

### Study selection and data extraction

Study selection was performed by three independent reviewers (AL, CS, GM) and disagreements were resolved by a fourth party (KH) [34]. Titles and abstracts of retrieved references were screened for inclusion and full texts of potential articles were analysed for meeting the inclusion criteria.

Data extraction was performed by three independent reviewers using a standardised form (AL, MS, GM). Definitions of injurious falls and methods used to record injurious falls were extracted from papers and classified according to subcategories. Frequencies of the definitions and methods used were documented as absolute (number of articles = n) and relative (number of articles using a specific definition or method/number of articles included in the review * 100) values. Where possible, the number of falls and injurious falls was extracted from the papers and the proportion of injurious falls to all falls (number of injurious falls/number of all falls * 100) was calculated. Relationship between the type of injurious fall definition used in studies and the proportion of injurious falls reported was evaluated by descriptive analysis. To measure the variability of the proportion of injurious falls between studies, the coefficient of variation [35] [(CV = (standard deviation/mean) × 100)] was calculated. The CV expresses the percentage variation between articles, thereby providing an indicator of agreement with respect to the proportion of injurious falls. Higher CVs indicate less agreement between studies.

## Results

### Selection of articles

**Figure 1:**
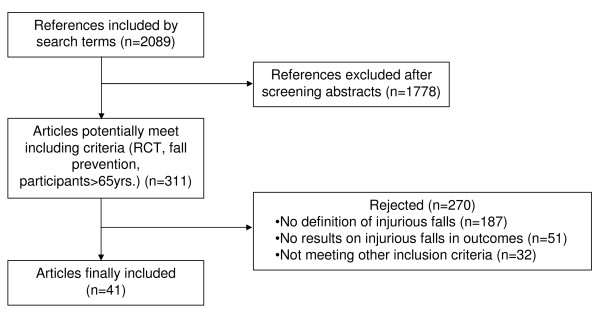
**Flowchart of the process of literature search and extraction of RCTs meeting the inclusion criteria**.

The search yielded 2089 articles, 1778 were excluded on initial screening as not fulfilling entry criteria, thus 311 were potentially appropriate for inclusion in the review (Figure 1). Of these 311 papers, 270 were excluded, because on closer inspection they did not provide a definition of injurious falls or did not report on injurious falls or did not meet other inclusion criteria. Forty-one articles were finally included in the review.

### Injurious fall definition

Definitions of injurious falls used in the articles included in this review are shown in Table 1. There was considerable heterogeneity in defining injurious falls and no definition stood out as the gold standard. Three main categories of definitions were found: (1) definitions based on symptoms (n = 16, 39%); (2) combined definitions based on symptoms and healthcare use (n = 19, 46%); (3) definitions based on healthcare use (n = 6, 15%). The most frequent type of definition that has been used similarly in different articles (n = 6, 15%) originates from a study by Campbell et al. [36]. This definition sub-classifies falls according to severity (serious, moderate) by using both symptomatic features (fractures, bruising, sprains, cuts, abrasions, reduction in physical function) and healthcare use (hospital, any wounds needed stitches, medical help). Referencing for this definition was inconsistent as some papers gave other primary sources [37] and some no reference at all [36,38,39].

**Table 1:** Types of definitions used to describe injurious falls in RCTs

Type of definition, n (%)	Description of definition
**Based only on symptoms, 16 (39%)**	

Fractures only, 6 (14%)	• Hip fractures and non-hip fractures [40] • All fractures [41-45]

AIS definition, 3 (7%)	• AIS scores: 1 = minor injury, 2 = moderate injury, 3 = severe, non-life-threatening injury, 4 = severe, life-threatening injury, 5 = critical injury [46,47] • Adapted AIS Scale ranging from "no injury, no pain" (level 1) to "fractures of hip, leg, or skull" (level 7) [48]

Other definitions, 7 (17%)	• Bruises, strains, cuts and abrasions, back pain and fractures [49,50] • Any detectable residual adverse physical change persisting beyond 1 hr after the fall [51] • Fractures or soft tissue injuries [52] • Cuts and bruises, fractures or other trauma [53] • If participants indicated that they were injured by the fall, irrespective of the severity of the injuries [54] • Fracture, dislocations, and organ and soft tissue trauma [55]

**Based on symptoms and healthcare use, 18 (44%)**	

FICSIT definition, 4 (10%)	• Original FICSIT definition: Fractures, head injuries requiring hospitalisation, joint dislocations, sprains and lacerations requiring suturing [56,57] • Modified FICSIT definition: fractures, other head injuries with altered consciousness, joint dislocations or sprains, or sutured lacerations [58]; Fractures, head injuries requiring hospitalisation, joint dislocations, severe sprains and lacerations requiring suturing [59]

Definition according to Campbell et al. [36], 6 (15%)	• Serious: fracture or admission to hospital or if any wounds needed stitches (sutures); moderate: bruising, sprains, cuts, abrasions, or a reduction in physical function for at least three days, or if the participant sought medical help [36-39,60,61]

Other definitions, 8 (20%)	• dislocations, injuries of chest, abdomen, or pelvis; open wounds requiring suturing; injuries to blood vessels; crushing injuries; and injuries to nerves and spinal cord [62] • Injury: any sequelae relating from a fall; serious injury: sprains, joint dislocation, hip fracture, other fracture, transfer to acute hospital, urgent physician visit, radiological examination [63] • Injury falls: any fracture, strain, sprain, laceration, or persistent pain (more than seven days); Medical care falls: any fall for which medical care was sought; Fracture falls and hospitalised falls [64] • Fractures, dislocations and soft tissue injuries needing suturing and even more severe injuries [65] • Fractures, head injuries, sprains, bruises, scrapes, or other serious joint injuries, or if the participant sought medical care [66] • Medical attention falls were coded ranging from no injury (0) to broken bone (4) [67] • Cut, scrape, gash, bruise or fracture; a head injury resulted or where the fall resulted in hospitalisation [68] • Fractures and hospital admission [69]

**Based only on healthcare use, 7 (17%)**	

Healthcare use definition, 7 (17%)	• Medical treatment [70,71] • Hospital visits or admission for the treatment of a fracture or suspected fracture [72] • Medical care [73] • Falls requiring medical attention [74] • Doctor's or hospital attendance [75] • General practitioner or emergency department or admission to the hospital [76]

n = number of articles used a specific definition; AIS = Abbreviated Injury Scale [77]; FICSIT = Frailty and Injuries: Cooperative Studies of Intervention Techniques [78]

Four articles (10%) used the definition of the Frailty and Injuries Cooperative Studies of Intervention Techniques (FICSIT) collaboration, with two studies [56,57] using the original definition and two using a modified version ("head injuries with altered consciousness" instead of "head injuries requiring hospitalisation" [58]; "severe sprains" instead of "sprains" [59]). Three articles (7%) defined injurious falls according to the Abbreviated Injury Scale (AIS) [77] using a severity score based on medical symptoms (ranging from low-level injuries that did not require medical attention to high-level injuries which necessitated medical care). One paper [48] used a modified version of the AIS.

Six included papers (14%) defined injurious falls only by fractures whereupon one study [40] defined hip fractures and non-hip fractures and five did not specify the type of fracture [41-45]. Seven articles (14%) defined injurious falls only by healthcare use by either using unspecific terms such as "medical treatment" [70,71] or "medical care" [73] or specific definitions such as "hospital visits or admission for the treatment of a fracture or suspected fracture" [72].

A considerable number of articles (n = 15, 37%) used heterogeneous definitions by either defining symptoms (n = 7, 17%) or by combining symptoms and healthcare use (n = 8, 20%). These types of definitions ranged from single sentences including a variety of symptoms (e.g. bruises, strains, cuts and abrasions, back pain and fractures [49,50]) regardless of severity to multiple-level definitions categorising injurious falls according to five levels of severity (from no injury [level 0] to broken bone [level 4] [67]). The majority of studies (n = 30; 73%; [36,38-45,49-55,58,61-64,67-70,72-76]) did not provide a reference for the definition of an injurious fall.

### Methods of collecting injurious falls data

Table 2 details the methods of collecting injurious falls data used in RCTs. There was considerable heterogeneity in reporting systems and the time period over which information was collected. Three main methods of collecting falls data were found in the articles: (i) prospective reporting systems using calendars, postcards or diaries (n = 33, 80%); (ii) retrospective reporting systems using telephone interview or postal questionnaire (n = 30, 73%); (iii) medical records (n = 18, 44%). Prospective registration systems requested immediate return of the data, or return at specified time points ranging from one week to three months. Studies used prospective reporting systems often followed up with data collection by secondary data capture mechanisms where by telephone calls (82%), hospital records (24%), records from nursing homes, physicians or emergency departments (15%) or radiological records (12%) were used.

**Table 2:** Methods of collecting injurious falls data

**Ref**.	Prospective			Retrospective	Medical record
	Method	Recording	Reporting		
**Fracture definition**					
[40]	Calendar	daily	not specified	-	Radiological
[44]	Calendar	weekly	not specified	Postal Questionnaire	-
[41]	Diary	after fall	every three weeks	-	Hospital, Other^4^
[45]	Calendar	daily	monthly	Phone^1,2^	-
[42]	Diary	daily	Every two month	Phone^3^	Radiological
[43]	Calendar	daily	monthly	Phone^1,2^	Radiological
**AIS definition**					
[46]	not specified	daily	after fall	-	Hospital
[47]	-	-	-	-	Hospital
[48]	Diary	daily	monthly	Phone^3^	-
**FICSIT definition**					
[56]	Calendar	daily	monthly	Phone^1,2^	-
[57]	Calendar	daily	monthly	Phone^3^	Hospital, Other^4^
[58]	-	-	-	-	Hospital, Radiological, Other^4^
[59]	Calendar	daily	monthly	Phone^1,2^	-
**Campbell et al. Definition**					
[36]	Calendar	daily	monthly	Phone^1,2^	Hospital
[60]	Calendar	daily	monthly	Phone^1,2^	Hospital
[38]	Calendar	daily	monthly	Phone^2^	Other^4^
[37]	Calendar	daily	monthly	Phone^1,2^	-
[61]	Calendar	daily	monthly	Phone^2^	Hospital, Other^4^
[39]	Calendar	daily	monthly	Phone^1,2^	-
**Health care use definition**					
[71]	-	-	-	-	Hospital, Other
[70]	Diary	daily	monthly	Phone^1,2^	-
[72]	Calendar	weekly	monthly	Phone^1,2^	-
[73]	Calendar	daily	monthly	Phone^3^	-
[74]	-	-	-	Phone^3^	-
[75]	Diary	daily	After 3,6 and twelve month	Phone^3^	-
[76]	Diary	daily	monthly	Phone^3^	Hospital
**Other definitions**					
[50]	-	-	-	Phone^1^, Postal Questionnaire	-
[49]	Calendar	daily	monthly	Phone^1,2^	-
[51]	Postcard	weekly	monthly	Phone^1^	-
[52]	-	-	-	-	Hospital, Radiological, Other^4^
[53]	Diary	daily	monthly	-	-
[54]	Calendar	daily	monthly	Phone^1,2^	-
[55]	Calendar	daily	monthly	Phone^3^	-
[62]	Diary	daily	monthly	Phone^2,3^	-
[63]	Calendar	daily	monthly	-	Radiological
[64]	Calendar, Postcard	daily	every three month, after fall	Phone^2, 3^	-
[65]	-	-	-	Phone^3^	Hospital, Other^4^
[66]	Calendar	daily	every three month	-	Hospital, Other^4^
[67]	Diary	daily	every two weeks	Phone^1^	-
[68]	Calendar	daily	monthly	-	-
[69]	Postcard	-	monthly	Phone^1^	-
Method applied in n (%^5^)of studies:	Calendar: n = 22 (54%) Postcard: n = 3 (7%) Diary: n = 9 (22%) not specified: n = 1 (2%) not applied (-): n = 7 (17%)	daily: n = 29 (71%) weekly: n = 3 (7%) after fall: n = 1 (2%) not applied (-): n = 8 (20%)	weekly: n = 2 (5%) monthly: n = 29 (71%) after fall: n = 2 (5%) not specified: n = 2 (5%) not applied (-): n = 7 (17%)	Phone: n = 29 (71%) Postal Questionnaire: n = 2 (5%) not applied(-): n = 11 (27%)	Radiological: n = 6 (15%) Hospital: n = 13 (32%) Other: n = 9 (22%) not applied (-): n = 23(56%)

Given are the methods of collecting injurious falls data in the articles included in the review. n = number of studies; AIS = Abbreviated Injury Scale; FICSIT = Frailty and Injuries: Cooperative Studies of Intervention Techniques

^1^if calendar, diary, or postcard not returned; ^2^following occurrence of a fall; ^3^regular phone call; ^4^records from nursing homes, physicians or emergency departments; ^5^calculation of percentages: number of articles used a specific method/total number of articles * 100, percentages may sum up to more than 100 percent as some studies used multiple methods

Studies using the Campbell et al. definition [36-39,60,61] consistently collected data by prospective daily recording with calendars, monthly reporting, and telephone interview follow up. In contrast there was little consistency in methods of data collection among studies that used other types of definitions (FICSIT, fracture, AIS, health care use, other).

Three studies (7%) [47,52,58] collected data on injurious falls only by the use of medical records whereupon information was gathered from hospitals [47,52,58], nursing homes [47,52,58], radiology departments [52,58], emergency departments [52,58], or physicians' records [52,58].

### Proportion of injurious falls to all falls

For 27 (65.9%) studies the proportion of injurious falls to all falls was computed (Table 3). For 13 (31.7%) studies [44,45,50-53,57,58,65,69,71-73] calculation of the proportion of injurious falls is not possible as data on the number of falls and/or number of injurious falls are not available. Two papers [36,39] use the same data, one paper [39] is therefore not included in Table 3.

**Table 3:** Falls and proportion of injurious falls as reported in the RCTs

**Ref**.	Sample size, n	All falls, n	Proportion of injurious falls to all falls, % (n)				
			Total	Fracture	Serious	Moderate	Healthcare use
			**Definitions based only on symptoms**				
			Fracture definition				
[40]	981	1527	4.5% (68)	2.1%(32)^1 ^ 2.4%(36)^2^	-	-	-
[41]	196	460	3.6% (7)	3.6%(7)^3^	-	-	-
[42]	242	275	11.3% (31)	11.3%(31)^3^	-	-	-
[43]	2.256	5.404	4.6% (246)	4.6%(246)^3^	-	[59.2% (3.198)]^8^	-
			AIS definition				
[46]	384	619	23.4% (145)	2.4% (15)^4^ 2.9%(18)^5^	-	18.1%(112)^9^	-
[47]	199	78	28.2% (22)	2.6%(2)^1^ 2.6%(2)^2^	-	23.1%(18)^7^	-
[48]	230	89	56.2% (50)	1.1%(1)^1^ 4.5%(4)^2^	14.6%(13)^6^	36%(32)^10^	-
			Other definition				
[49]	597	510	63.5% (324)	-	-	-	-
[54]	186	322	39,4% (127)	-	-	-	-
[55]	597	957	48,0% (459)	-	-	-	-
[62]	360	367	8.2% (30)	1.9%(7)^3^	6.3% (23)	-	-
			**Definitions based on symptoms and health care use**				
			FICSIT definition				
[56]	288	238	24.0% (57)	-	24.0% (57)	-	-
[59]	291	258	35.7% (92)	-	12.0% (31)	-	23.6%(61)^11^
			Campbell et al. definition				
[36]	233	240	45.8% (110)	-	10.4% (25)	35.4% (85)	-
[60]	152	358	40.5% (145)	-	9.5% (34)	31.0% (111)	-
[38]	391	443	51.5% (228)	-	6.8% (30)	44.7% (198)	25.5%(113)^11^
[37]	312	584	55.8% (326)	-	3.6% (21)	52.2% (305)	-
[61]	240	189	48.1% (91)	-	5.8% (11)	42.3% (80)	23.3% (44)^11^
			Other definition				
[63]	547	1299	40.3% (523)	-	4.2% (54)	-	-
[64]	3.182	3.814	30.5% (1.163)	5.1%(193)^3^	-	-	14.6% (558)^11^ 2.9%(111)^12^
[66]	188	111	21.6% (24)	-	2.7% (3)	18.9% (21)	17.1%(19)^12^
[67]	70	546	14.6% (80)	0.4%(2)^1^ 3.3%(18)^2^	-	-	-
[68]	1.090	1.448	55.5% (804)	-	-	-	8.6% (124)^13^
			**Definitions based only on health care use**				
[70]	27	23	30.4% (7)	-	-	-	30.4% (7)^14^
[74]	3.139	3.776	21.7% (821)	-	-	-	21.7% (821)^15^
[75]	349	224	13.5% (47)	-	-	-	9.2%(32)^16^ 4.3%(15)^17^
[76]	392	820	12.2% (100)	-	-	-	12.2%(100)^12,16,17^
**Range:**			**3.6-63.5%**	**0.4-11.3%**	**2.7-24.0%**	**18.1-52.2%**	**2.9-30.4%**
**CV:**			**50.1%**	**51.5^18^**	**68.0%**	**35.4%**	**46.8%**

Displayed are falls (number) and injurious falls (proportion = injurious falls/falls * 100; number) as reported in the RCTs. Injurious falls data are given for the following categories: total, fracture, serious, moderate, healthcare use

n = number; AIS = Abbreviated Injury Scale; FICSIT = Frailty and Injuries: Cooperative Studies of Intervention Techniques. CV = Coefficient of variation [(SD/mean) × 100]) as a measure of the variability (%) of injurious falls outcomes between studies. ^1^Hip fracture; ^2^other fracture (except hip); ^3^fracture type not specified; ^4^hip or other femoral fractures; ^5^vertebral or wrist fracture; ^6^pain, injury, medical help required; ^7^Injuries; no fracture; ^8^bruise, abrasion or muscle injury without fracture, results are not included in analysis as only fractures were defined; ^9^superficial wounds and bruises; ^10^pain, minor injury, friends help needed, Bruises and no help needed; ^11^medical care; ^12^hospitalised falls; ^13^medical treatment; ^14^hospitalisation, emergency department visits or physician visits; ^15^medical attention; ^16^general practitioner surgery; ^17^accident and emergency department; ^18^total fracture incidence per study

For the following reference calculation of the proportion of injurious falls is not possible as data on the number of falls and/or number of injurious falls are not available: [44,45,50-53,57,58,65,69,71-73]. Reference [39] uses the same data as [36] and is therefore not included in the table

Calculation of the proportion of falls which are injurious allows a comparison of injurious falls outcomes across studies. A considerable range (3.6%-63.5%) in the proportion of injurious falls is apparent. The proportion appears to be related to the type of definition used: lowest proportions are found in studies that defined injurious falls as fractures only (0.4%-11.3%), studies using multi-level definitions such as the AIS report higher proportions (23.4%-56.2%) and the highest proportion (63.5%) is found if a single definition had been used which included a range of symptoms (e.g. back pain, bruises, strains, cuts and abrasions, and fractures [49]).

For the proportion of total injurious falls reported across studies, variation is high (CV = 50.1%). Considerable variation between studies is also apparent for subcategories of injurious falls in terms of fractures (CV = 51.5%), serious (CV = 68.0%) and moderate (CV = 35.4%) falls. However, variation is related to the standardisation of definition and outcomes: for the group of studies using versions of the Campbell et al definition, variation in the proportion of all injurious falls is considerably lower (CV = 12.0%) compared to variation of other studies included in the analysis (CV = 66.3%). The same result is obtained for the subcategories: across studies which applied the Campbell et al. definition a lower variation is apparent for proportion of serious (CV: Campbell et al.: 38.3% vs. other studies: 75.2%) and moderate (CV: Campbell et al.: 20.1% vs. other studies: 35.5%) injurious falls compared to all studies included.

In those studies using healthcare definitions (n = 10, 24.4%) the proportion of injurious falls varies substantially (2.9-30.4%) leading to a high CV of 46.8%. Lowest proportion is apparent for injurious falls defined as "hospitalisation" (2.9% [64]), whereas high proportions of injurious falls are apparent if unspecific "medical care" definitions are used (14.6-23.6% [38,59,61,64]) (Table 3).

### Methods of summarising injurious fall outcomes

Table 4 details the methods used to summarise data on injurious falls in identified articles. The way data are summarised differs across papers. The most frequently reported summary statistic is the number of participants sustaining an injurious fall (n = 17, 41%) and the number of injurious falls (n = 17, 41%).

**Table 4:** Methods of summarising injurious fall outcomes

Data reported	Articles n (%)	Reference
Number of participants sustaining injurious falls	17 (41%)	[36,38,40,41,47,51,53,55,59,63,65-67,70,72,73,76]
Sustaining medical care falls	5 (12%)	[53,59,69,74,75]
Sustaining fracture falls	11 (27%)	[40-44,46,47,53,55,69,75]
Number of injurious falls	17 (41%)	[43,46,48,49,52,54,55,58,59,61,63,64,67,68,71,73,76]
Medical care falls	11 (27%)	[38,39,56,59,61,64,66,68,74-76]
Fracture falls	11 (27%)	[40,42,43,45,52,58,62,64,67,71,76]
Falls requiring hospital admission	3 (7%)	[58,59,64]
Injury rate	4 (10%)	[40,52,54,65]
Injurious fall rates	8 (20%)	[37-39,56,58,61-63]
Mean number of injurious falls per participants	5 (12%)	[37,40,49,63,67]
Injurious falls/person time	11 (27%)	[40]^1 ^,[54]^1^, [62]^5^, [38]^4^,[61]^2^,[63]^4^,[68]^4^,[58]^4^,[52]^4^,[2]^2^,[39]^4^
Serious injury/person time	3 (7%)	[37,38,63]^2^
Moderate injury/person time	2 (5%)	[37,38]^2^
Medical care falls/person time	1 (2%)	[56,68]^4^
Soft tissue/person time	1 (2%)	[52]^1^
Hip fracture/person time	2 (5%)	[40,52]^1^
Other fracture/person time	2 (5%)	[40,52]^1^
Time to injurious fall event	4 (10%)	[43,48,57,76]

Methods of fall documentation used in the articles. n = number of studies; calculation of percentages: number of articles used a specific method/total number of articles * 100; ^1^number/1000 persons year; ^2^number/persons year; ^3^number/10 persons year; ^4^number/100 persons year

Some papers report on injurious falls by specifying the number of participants sustaining medical care falls (n = 5, 12%) and/or the number of medical care falls (n = 11, 27%). A few articles (n = 3, 7%) report on the number of falls requiring hospital admission. Fracture falls are specified in some papers as number of events (n = 11, 27%) and in some as number of participants (n = 11, 27%). Categories of injurious falls partly overlap as some studies report on both less specific types of injurious falls (e.g. medical care falls) and more specific types of injurious falls (e.g. fracture falls) whereas other studies only report one category.

Beside absolute values, several articles report rates of injurious falls or fall-related injuries. Calculation of rates was inconsistent. Some studies calculated the mean number of injurious falls per participant (n = 5, 12%). A number of studies calculated a particular relationship between a numerator and a denominator, where the denominator included time measurement. Different types of numerators (severity of injury, type of injury such as fracture or soft tissue) and denominators (resident year, 10 resident years, 100 person years, 1000 person years) had been used for calculation (Table 4).

## Discussion

The results of this review highlight that existing fall prevention RCTs substantially vary in their methods of defining, collecting, and reporting injurious falls. No method has to date been implemented as a designated standard. Instead, a variety of approaches to defining and categorising injurious falls are identified. The use of different approaches leads to substantial differences in study results and thus limits the comparability of findings, and makes interpretation difficult.

### Injurious fall definition

A large variety of definitions, in a number of cases not referenced, are identified in the present review. Some studies defined only specific injuries, such as fractures, while others collapsed multiple symptoms related to injurious falls into a single definition. The proportion of injurious falls to all falls reported substantially increases if several symptoms of different grades of severity are included into a single definition. For example, Lord [49] defined bruises, strains, cuts and abrasions, back pain and fractures and reported 63.5% of all falls were injurious. In contrast, Becker included only radiologically confirmed fractures resulting in 4.5% being reported as injurious falls. Although some of the differences between studies might be explained by the different populations and settings, the high proportion of injurious falls in the Lord study is most likely due to the inclusion of falls that caused minor injuries [49].

Inconsistent definitions with respect to healthcare use limit the comparability of RCTs, e.g. studies which used the FICSIT definition [56,59] included injurious falls predominantly requiring medical care and thus reported lower proportions of injurious falls (24-35.7%) compared to a study [49] which uses definitions based on multiple symptoms (63.5%) not all necessitating medical care use (e.g. abrasion, back pain).

Various definition of healthcare use can be identified ranging from specific interventions such as "suturing" to unspecific terms such as "medical treatment" or "urgent physician visits"and this exacerbates problems in comparing comparability of study outcomes. Methodological problems in injury research with respect to healthcare use have been discussed elsewhere [32]. If medical care services are used as a measure of injury, then the definition of injury becomes entangled with service configuration and policy issues, the type and availability of care and factors that influence care-seeking behaviour, including personality, pain tolerance, and anxiety [32].

Grouping injuries by severity, as found in several studies included in this review, is important; however, the use of different schemes can lead to confusion and makes it difficult to compare findings [32]. Authors who used differentiated scales for severity classification such as the AIS (seven categories) [48] summarised single categories into one (e.g. 4-7) either in order to facilitate statistical analysis or to compare results with other studies that used only one or two categories. However, the way severity categories were collapsed differ between studies and hamper the comparability of outcomes. A small change in the definition of injury severity can substantially influence the reported proportion of injurious falls as indicated by the following example: use of the expression "severe sprains" in the FICSIT definition resulted in 12% of falls being serious injurious falls [59]. In contrast 24% of falls were serious injurious falls were reported if "sprains" (without the word "severe") were defined [56].

Overall, the adverse impact of the variability in definitions and categorisation systems is reflected by the substantial variation of the proportion of injurious falls across studies. High CVs were found for the proportion of all injurious falls as well as for subgroups or fracture, serious, moderate and healthcare falls. However, one of the major findings of the present review is that a strict standardisation of the definition could substantially enhance comparability of studies. This is reflected by the group of RCTs which defined injurious falls according to Campbell et al. [36-39,60,61]. Whereas high variation had been obtained between outcomes of all RCTs included in this review, variation was considerably lower across those studies which applied the standardised Campbell et al. definition. This highlights the importance of using a standardised definition in future.

### Methods of collecting injurious falls data

The high variation in proportion of injurious falls found in the present study may not only be due to the varying definitions, but also to different assessment methods. Several studies did not record injurious falls prospectively [47,50,52,58,65,71,74] or did not follow up prospective data collection to verify information on injurious falls, e.g. by phone call [40,41,46,47,53,63,66,68]. In addition, periods of recording data by the study centre substantially varied (from three weeks to three months) between studies. Long delay between event and data recording might limit the verification of injurious falls information. Thus, in particular due to short recovery periods of soft-tissue injuries [13] and memory deficits in the elderly [79] prospective daily recording in combination with short latency periods for reporting information to the study centre is essential to gather valid information about injurious falls [13].

Interestingly, we found that those RCTs which had higher consistency in the proportion of injurious falls (RCTs using the Campbell et al. definition) used similar methods for data collection suggesting that a standardised assessment enhances the comparability of results.

Some variation in proportion of injurious falls might be related to the limited standardisation of medical records. Medical reports have been recommended for confirming prospective injurious falls data and verifying severity of injury [78]. Radiological confirmation of fractures has been requested as a gold standard [13]. However, only 44% of the authors used medical reports as a data source and radiological confirmation of fractures was reported in only 15% of the RCTs included in the review. Accuracy of self-report of fracture is questionable since its agreement with radiologic diagnosis is limited [80].

Studies differed considerably with respect to the type of patient reports used. Some studies included nursing home reports [41,52,58] whereas others only included reports of physicians [38,46,57,60,63] or emergency departments [46,57,63]. As records can differ in validity [13] this might explain some of the variability, particularly in those studies [47,52,58] which used medical reports as a primary data source.

### Injurious falls documentation

The way data are summarised differs among articles. Several studies reported on the number of injurious falls or on injurious fallers, whereas others specified fractures or reported on medical care falls. Selection of outcomes depends on the predefinition of specific endpoints such as serious injuries or fractures. For a number of studies the calculation of the proportion of injurious falls was not possible as data on the number of falls and/or injurious falls were not given and only fallers, injurious fall rates or fall rate ratios were reported. Varying numerators (fracture, serious, moderate, healthcare use) and different time denominators (1-1000 years) additionally exacerbate the comparability of outcomes as recently highlighted standardised documentation of injuries is a crucial issue for injury epidemiology and should be implemented in future studies [81].

### Development of a standardised methodology for future RCTs

To date no standardised methodology exists for defining, assessing, and reporting injurious falls in RCTs, and thus needs to be developed for future research.

The most robust measure of injurious falls is considered to be peripheral fracture rate, verified by radiological evidence [13,29,78]. However, definition of fractures as primary endpoint requires large sample sizes [29,82]. By way of example, we determined sample sizes in order to detect statistically significant differences in outcomes related to falls and injurious falls (Table 5). The calculations demonstrate that, for instance, in a study population with a hip fracture incidence of 3% (medium risk) [83] a sample close to 42.000 study participants would be needed to observe a significant effect of an intervention if it reduced the hip fracture incidence by 15% (significance level 5%, power 80%). Based on this assumption no study in the present review met the requisite sample size calculation disallowing any statement on effectiveness of interventions with respect to falls resulting in hip fracture.

**Table 5:** Hypothetical examples of sample size requirements for RCTs of injurious falls prevention by risk group

Outcome	Incidence in study population	Required sample size
**Low risk (25% lower than intermediate risk)**		
Fallers^1^	37.50%	2246
≥ 1 injurious falls	15.00%	7408
≥ 1 fractures	3.75%	33222
≥ 1 hip fractures	2.25%	56168
**Intermediate risk**		
Fallers^1^	50.00%	1386
≥ 1 injurious falls	20.00%	5258
≥ 1 fractures	5.00%	24618
≥ 1 hip fractures	3.00%	41828
**High risk (25% higher than intermediate risk)**		
Fallers^1^	62.50%	868
≥ 1 injurious falls	25.00%	3966
≥ 1 fractures	6.25%	19456
≥ 1 hip fractures	3.75%	33222

Assumptions for sample size calculation: effect size -15% with respect to all outcomes, statistical power 80%, significance-level 5%, two-sided tests, not adjusted for multiple testing.

^1^Fallers reflect those study participants who experience any fall (injurious or not injurious) during the study period

Including a range of fall-related injuries into one definition substantially increases the incidence of injurious falls and thus requires smaller sample sizes to achieve adequate statistical power. However, if several symptoms are included then a proper definition with respect to severity and the use of medical care is essential for comparability of results. Established scales such as the AIS provide a differentiated severity scoring (7 categories); however, its utility for population-based research is limited due to the time-consuming assignment of scores [84]. In contrast, categorising serious and moderate injurious falls according to the definition of Campbell et al. is feasible, even in large scale studies. It is the most frequent type of definition that has been used consistently in different articles (n = 6, 15%) included in the review. A meta-analysis, including RCTs and controlled trials, has been successfully conducted on the basis of the Campbell et al. definition [24].

**Figure 2:**
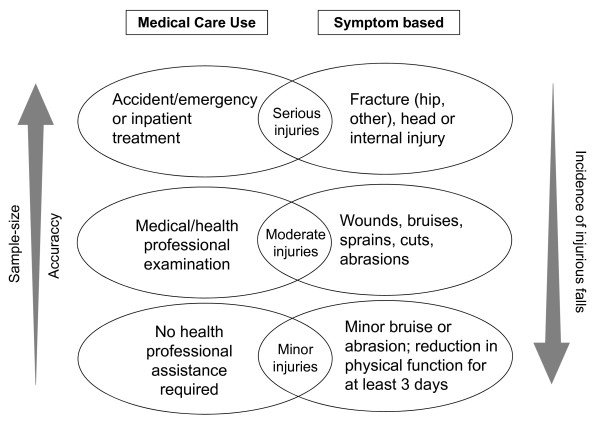
**Injury categories of the newly developed system are defined by both symptoms and medical care use**. Definition of serious injuries represents the endpoint with highest accuracy but requires largest sample size due to low incidence of these falls. Definition of moderate and/or minor injuries requires smaller sample sizes but reduces accuracy of data (illustrated by left and right arrow).

According to our findings, a comprehensive, standardized system for categorising and defining serious, moderate and minor fall-related injuries by both symptoms and medical care use is recommendable for future RCTs of fall prevention (Table 6, Figure 2). Categories can be chosen for defining specific types on injurious falls depending on the research question and sample size calculation requirements. Each of the categories can be used independently as it is characterised by a standardised definition. Ideally, the injuries in each category should be reported even if a specific study is not powered to detect effects. Reporting all injuries will prevent an outcome bias and the data will be available for future meta-analysis. Data of medical care can be used for focus on cost calculation. A fall-related injury should be classified by an independent person, blind to group allocation.

**Table 6:** Standardized system for categorizing and defining fall-related injuries

Category	Definition
a - serious injury	medically recorded fracture, head or internal injury requiring accident and emergency or inpatient treatment
b - moderate injury	wounds, bruises, sprains, cuts requiring a medical/health professional examination such as physical examination, x-ray, suture
c - minor injury	minor bruises or abrasions not requiring health professional assistance; reduction in physical function (e.g. due to pain, fear of falling) for at least three days.
d - no injury	no physical injury detected

Accuracy of moderate and minor injurious falls data is lower as the definition of injury becomes entangled with the type and availability of care and factors that influence care-seeking behaviour, including personality, pain tolerance, and anxiety [32] (Figure 2). However, research on falls causing minor injuries has been highlighted as an important issue as they have also serious consequences such as depression, fear of falling and activity restriction [14].

Injurious falls data should be collected by prospective daily recording, a minimum of monthly reporting, and telephone or face-to-face interview as recommended in a consensus paper on fall prevention studies [13]. We recommend standardised documentation of injurious falls data as number of injurious falls, number of people sustaining injurious falls, injurious falls rate per person-year of follow-up, and number of people sustaining multiple events [13]. Along with a standardised statistical analysis indicating the absolute risk difference between groups a common data set will improve comparability of future RCTs.

### Limitations

Only RCTs in fall prevention were included in the present review and therefore it reflects the methodological status in this specific research area. We are aware that by this pre-selection relevant data, including epidemiological sources may not have been considered. We also note that our inclusion only of papers that defined their terms may have resulted in exclusion of papers where the outcome might be considered self-evident, for example we may have underreported the number of papers reporting fracture if fracture was not defined as an injury.

## Conclusion

To date, no method has been defined as an international standard for RCTs in the field of fall prevention. Defining serious and moderate injurious falls according to Campbell et al. is the most frequently used methodology in RCTs to date and allow a comparison of study results. On the basis of this methodology we developed a standardised system for defining different categories of injuries which is feasible also in large scale studies. We recommend use of this system in future studies to reach a consensus on injurious falls methodology.

### What is already known on this subject

• The combination of single randomised controlled trials in meta-analyses may help to generate evidence based data on specific prevention strategies to reduce injurious falls.

• The standardisation of the assessment methodology and case definition represents a major precondition for the conduction of meta-analyses.

• International guidelines provide recommendations for the standardisation of falls methodology; however, injurious falls have not been targeted.

### What this study adds

• The definitions and methods used to measure and document injurious falls substantially vary in existing randomised controlled trials on fall prevention and thus hampering the comparability of study results and the conduction of meta-analyses.

• Our results highlight that the use of a standardised definition of injurious falls leads to a higher concurrence in study outcomes.

• Based on our results we recommend use of a standardised methodology in future randomised controlled trials including a comprehensive system for categorising and defining injurious falls and standardised methods of collecting and reporting on injurious falls data.

## Competing interests

The authors declare that they have no competing interests.

## Authors' contributions

MS: development of study concept and design, preparation of manuscript, interpretation of data, statistical analysis; AL: literature search, study selection, interpretation of data, preparation of manuscript, development of study design; CS: literature search, study selection, critical revision of manuscript; RRM: literature search; GM: literature search, study selection, critical revision of manuscript; CT: critical revision of manuscript; KH: study selection, development of study concept and design, critical revision of manuscript. All authors including PO contributed to the interpretation of data, drafting the article and final approval of published version. All authors read and approved the final manuscript.

## Pre-publication history

The pre-publication history for this paper can be accessed here:

http://www.biomedcentral.com/1471-2288/12/50/prepub
